# 67702 At-Home Screening Tool for Anosmia

**DOI:** 10.1017/cts.2021.764

**Published:** 2021-03-30

**Authors:** Shruti Gupta, Dorina Kallogjeri, Jay F Piccirillo

**Affiliations:** Washington University School of Medicine

## Abstract

ABSTRACT IMPACT: By developing and validating a simple and cost-effective at-home screening tool for loss of smell, we can efficiently detect infection with COVID-19, neuropsychiatric disease such as Alzheimer’s, and post-operative smell loss. OBJECTIVES/GOALS: To develop and validate a feasible and cost-effective screening tool for olfactory dysfunction (OD) using common household items. METHODS/STUDY POPULATION: The study has two phases. In the Development phase, 120 participants with self-reported smell changes will complete a survey with a list of 45 household items to smell. Item reduction to develop the NASAL Short Smell Test will occur by measuring content validity, factor analysis, and internal consistency. In the Validation phase, 200 participants with self-reported smell changes will take the NASAL Short Smell Test at baseline and again at three weeks. In both phases, the validated University of Pennsylvania Smell Identification Test (UPSIT) will be used as the gold standard. Measures of performance as well as test-retest reliability and sensitivity to change will be measured. RESULTS/ANTICIPATED RESULTS: We anticipate that the majority of participants will have at least half of the items in their household and will report ability to smell for each. Measures of sensitivity, specificity, likelihood ratios, and UPSIT score correlations will allow us to evaluate performance of each item. Item reduction will allow us to create the NASAL Short Smell Test, in which a handful of common items will be used to create a screening tool for smell loss. The Validation phase will allow us to measure discriminative performance of this tool as well as test-retest reliability and sensitivity to change, which we expect to be at least comparable to the validated UPSIT. DISCUSSION/SIGNIFICANCE OF FINDINGS: Current tools for diagnosis of OD are costly, time-consuming, and often require a clinician to evaluate. The validation of the simple at-home NASAL Short Smell Test to screen for OD will allow us to detect infection with COVID-19, neuropsychiatric disease, or post-operative smell loss quickly and efficiently.

